# Exploring the association between feeding habits, non-nutritive sucking habits, and malocclusions in the deciduous dentition

**DOI:** 10.1186/s40510-015-0113-x

**Published:** 2015-12-18

**Authors:** Gabriela Mesquita Lopes-Freire, Abel Belizario Cahuana Cárdenas, José Enrique Espasa Suarez de Deza, Josep Maria Ustrell-Torrent, Luciana Butini Oliveira, Joan Ramon Boj Quesada JR

**Affiliations:** Department of Paediatric Dentistry, Faculty of Dentistry, University of Barcelona Hospitalet de Llobregat, Barcelona, Spain; Pediatric Dentistry and Orthodontic Service, Hospital Sant Joan de Déu, Barcelona, Spain; Department of Orthodontics, Oral Health and Masticatory System Group (Bellvitge Biomedical Research Institute) IDIBELL, Faculty of Dentistry, L’Hospitalet, Barcelona, Spain; Department of Pediatric Dentistry, School of Dentistry, Faculdade São Leopoldo Mandic, Campinas, SP Brazil

**Keywords:** Breastfeeding, Bottlefeeding, Malocclusion

## Abstract

**Background:**

This study aimed to explore the association between feeding habits, non-nutritive sucking habits, and malocclusions in deciduous dentition.

**Methods:**

A cross-sectional observational survey was carried out in 275 children aged 3 to 6 years and included clinical evaluations of malocclusions and structured interviews. Statistical significance for the association between feeding habits and the development of malocclusion was determined using chi-square and Fisher’s exact tests. In addition, odds ratio (OR) calculations were used for intergroup comparisons. Controlling for confounders was adjusted by excluding children with non-nutritive sucking habits.

**Results:**

The results indicated that there were no significant relationships between exclusive breastfeeding or bottlefeeding and the presence of any type of malocclusion (*p* > 0.05). There was also no significant association between breastfeeding or bottlefeeding duration and malocclusion (*p* > 0.05). In addition, it was observed that exclusive breastfeeding had a protective effect and diminished the risk of acquiring non-nutritive sucking habits (*p* = 0.001).

**Conclusions:**

There was no association between feeding habits and malocclusions in the deciduous dentition in this sample of children. Exclusive breastfeeding reduced the risk of acquiring non-nutritive sucking habits.

## Background

The sucking habits of infants are described in the literature as being of two types: non-nutritive and nutritive. Finger sucking, thumb sucking, and sucking on a pacifier (dummy, comforter) are considered non-nutritive sucking habits. Breastfeeding and bottlefeeding are considered nutritive sucking habits.

Several studies reported the association between feeding habits (breastfeeding and/or bottlefeeding), non-nutritive habits, and malocclusion [1–8]. The findings on associations between types and duration of feeding habits and malocclusion are conflicting. Moreover, confounder variables (presence of non-nutritive habits) were not performed in many previous studies.

The advantages afforded by breastfeeding have been reported in the literature and include the well-established immunological and psychological benefits, adequate weight gain for the baby, and the correct development of the oral structures involved in the action of sucking. A prospective birth cohort study from Brazil concluded that breastfeeding is associated with improved performance in intelligence tests 30 years later, and might have an important effect in real life, by increasing educational attainment and income in adulthood [9]. Previous studies showed that prolonged breastfeeding may have a protective effect on the development of malocclusions [1–7]. However, a recent systematic review of cohort studies concluded that the scientific evidence could not confirm the types of malocclusion associated with bottlefeeding or a proper period for breastfeeding in order to protect against malocclusion [10].

Observational surveys provide important information about the feeding practices and malocclusion status and contribute to the development of awareness among professionals as well as among the target population. It is important to investigate the presence of malocclusion in deciduous dentition because it is a public dental health problem in children in Spain. In addition, current recommendations for discontinuing non-nutritive sucking habits may be optimal in preventing habit-related malocclusions at the end of the primary dentition stage [11, 12].

Therefore, the aim of this study was to explore the association between feeding habits, non-nutritive sucking habits, and malocclusions in the deciduous dentition in a population of Spanish children.

## Methods

This study was approved by the Research Ethics Committee of the University of Barcelona and the Children Hospital of Barcelona and conducted in a day care center, CAP, Montcada i Reixac. The participants’ legal guardians gave positive consent on the day of the clinical examination.

A cross-sectional observational survey was carried out on boys and girls aged 3 to 6 years and included clinical evaluations of malocclusions and structured interviews.

All the children in the study met the inclusion criteria which included children of both genders aged 3 to 6 years; children exclusively in the deciduous dentition phase; agreement to participate in the clinical exam; all the normal numbers, sizes, and shapes of deciduous teeth; no major tooth destruction or reconstruction; children with no systemic diseases and/or neurological diseases; and parental questionnaires about the child’s habits.

Children were excluded from the study if their parents did not agree to their participation, they have syndromes or systemic problems affecting craniofacial growth, they have the presence of at least one permanent tooth, they have loss of mesial-distal diameter due to caries, and they have previous orthodontic treatment.

All clinical exams were performed by an experienced examiner, a PhD student (GMLF) who had previous experience in cross-sectional data. The child remained seated on a chair in front of the examiner. The examinations were performed under artificial light, using latex gloves and a disposable spatula. The clinical exam was performed with the aid of a disposable tongue depressor source. To ensure that natural occlusion was evaluated, the child was asked to open and close his/her mouth several times and to swallow saliva before the examination began. When necessary, the mandible was gently guided towards centric occlusion by the examiner.

The outcomes related to the children’s dental arch characteristics were examined in the three dimensions with the following criteria.

The transverse relation was measured by direct inspection in the presence of posterior crossbite or if the absence was considered normal occlusion. One of five separate relationships was recorded considering the following categories: normal relationship; posterior unilateral crossbite left side; posterior unilateral crossbite right side; bilateral posterior crossbite—both hemi-arches; and just one tooth was crossed. Posterior crossbite is considered present when, in occlusion, one or more of the maxillary deciduous canine or molars occluded lingual to the buccal cusps of the opposing mandibular teeth. Upper midline shift was registered if the midline was displaced by at least 1 mm. In addition, midline deviation data was also collected and the distance between the upper and lower midlines in the frontal plane was considered.

The vertical relation (relationship of incisors) was measured by direct inspection: one of three separate relationships was recorded in normal, anterior open bite, and overbite. Overbite was obtained by measuring the vertical distance between the upper and lower central incisor edges with the teeth in occlusion [13]. This distance was considered normal when the upper incisor covered the lower up to 3 mm and overbite for values greater than 3 mm. When there was no overlap between the upper and lower incisors, with a minimum space of 1 mm between both incisal edges, it was considered anterior open bite [14].

The sagittal interarch relationship was classified according to the deciduous canine relationship as angle class I, class II, or class III, with class I considered normal occlusion, class I canine and molars bilateral, or class I canine and molars unilateral, and class II or class III considered altered: class II bilateral, class II 1 (increased overjet), class II 2 (without overjet), class II subdivision (I o III), class II unilateral, others with no classification, and class III or anterior crossbite.

A questionnaire in the form of a structured interview was applied with mothers or guardians in order to find out about nutritive sucking habits (breastfeeding and bottlefeeding), non-nutritive sucking habits (pacifiers and finger sucking), and the presence of malocclusions. The data collected included the presence and the duration of non-nutritive sucking habits and, if the child had sucking, any type of non-nutritive sucking habits: pacifier-sucking habit and digit sucking.

Data analyses were performed using SPSS software 22.0. Data analysis included descriptive statistics (frequency distribution). Statistical significance for the association between the non-nutritive sucking habits and the development of malocclusion was determined using chi-square, and Fisher’s exact tests with odds ratio (OR) calculations were used for intergroup comparisons. Children with non-nutritive sucking habits were excluded from the analysis. The level of significance was set at 5 %.

## Results

The sample consisted of 275 children aged 3 to 6 years; 144 (52.4 %) were males and 131 (47.6 %) were females. Of 275 children, only 28 children were exclusively breastfeeding, and 247 children were breastfeeding/bottlefeeding. The presence of non-nutritive sucking habits was observed in 224 children (81.5 %).

The results presented in Table 1 indicated that there was no significant relationship between exclusive breastfeeding and the presence of any type of malocclusion OR 1.37 (confidence interval (CI) 0.34–5.51, *p* = 0.739). The results also indicated that there was no significant relationship between bottlefeeding and the presence of any type of malocclusion OR 1.35 (CI 0.31–5.96, *p* = 0.716) (Table 2). In addition, there was no significant association between breastfeeding or bottlefeeding duration and malocclusion (Tables 3 and 4).

**Table 1:** Relationship between exclusive breastfeeding and malocclusion

		Exclusive breastfeeding						
		No		Yes				
		*N*	%	*N*	%	OR	CI 95 %	*p* value
Transversal relationship	Malocclusion in transversal relationship	3	8.1	0	0.0	Infinity	NA	0.552
	Normal	34	91.9	14	100.0			
	Total	37	100.0	14	100.0			
Midline deviation	Malocclusion in midline	4	11.1	1	7.1	0.54	0.11–2.76	0.662
	Normal	32	88.9	13	92.9			
	Total	36	100.0	14	100.0			
Vertical relationship	Malocclusion in vertical relationship	6	16.2	3	21.4	0.71	0.15–3.33	0.692
	Normal	31	83.8	11	78.6			
	Total	37	100.0	14	100.0			
Sagittal relationship	Malocclusion in sagittal relationship	10	30.3	2	16.7	2.17	0.401–11.76	0.466
	Normal	23	69.7	10	83.3			
	Total	33	100.0	12	100.0			
Presence of malocclusion	Any type of malocclusion	13	40.6	4	33.3	1.37	0.34–5.51	0.739
	Normal	19	59.4	8	66.7			
	Total	32	100.0	12	100.0

**Table 2:** Relationship between exclusive bottlefeeding and malocclusion

		Exclusive bottlefeeding						
		Yes		No				
		*N*	%	*N*	%	OR	CI 95 %	*p* value
Transversal relationship	Malocclusion in transversal relationship	1	7.7	2	5.3	1.50	0.12–18.05	1.00
	Normal	12	92.3	36	94.7			
	Total	13	100.0	38	100.0			
Midline deviation	Malocclusion in midline	1	8.3	4	10.5	0.77	0.08–7.66	1.00
	Normal	11	91.7	34	89.5			
	Total	12	100.0	38	100.0			
Vertical relationship	Malocclusion in vertical relationship	1	7.7	8	21.1	0.31	0.04–2.78	0.42
	Normal	12	92.3	30	78.9			
	Total	13	100.0	38	100.0			
Sagittal relationship	Malocclusion in sagittal relationship	3	30.0	9	25.7	1.24	0.26–5.84	1.00
	Normal	7	70.0	26	74.3			
	Total	10	100.0	35	100.0			
Presence of malocclusion	Any type of malocclusion	4	44.4	13	37.1	1.35	0.31–5.96	0.716
	Normal	5	55.6	22	62.9			
	Total	9	100.0	35	100.0

**Table 3:** Relationship between bottlefeeding duration and malocclusion

		Bottlefeeding duration						
		>6–12 months		>12 months				
		*N*	%	*N*	%	OR	CI 95 %	*p* value
Transversal relationship	Malocclusion in transversal relationship	0	0.0	3	10.0	0	NA	1.00
	Normal	4	100.0	27	90.0			
	Total	4	100.0	30	100.0			
Midline deviation	Malocclusion in midline	1	33.3	3	10.0	4.5	0.31–65.67	0.33
	Normal	2	66.7	27	90.0			
	Total	3	100.0	30	100.0			
Vertical relationship	Malocclusion in vertical relationship	1	25.0	5	16.7	1.67	0.14–19.48	1.00
	Normal	3	75.0	25	83.3			
	Total	4	100.0	30	100.0			
Sagittal relationship	Malocclusion in sagittal relationship	2	50.0	8	30.8	2.25	0.27–18.93	0.584
	Normal	2	50.0	18	69.2			
	Total	4	100.0	26	100.0			
Presence of malocclusion	Any type of malocclusion	2	66.7	11	42.3	2.73	0.23–34.01	0.573
	Normal	1	33.3	15	57.7			
	Total	3	100.0	26	100.0

**Table 4:** Relationship between breastfeeding duration and malocclusion

		Breastfeeding duration						
		<6 months		>12 months				
		*N*	%	*N*	%	OR	CI 95 %	*p* value
Transversal relationship	Malocclusion in transversal relationship	2	11.1	0	0.0	Infinity	NA	0.229
	Normal	16	88.9	19	100.0			
	Total	18	100.0	19	100.0			
Midline deviation	Malocclusion in midline	2	11.1	2	10.5	1.06	0.13–8.47	1.00
	Normal	16	88.9	17	89.5			
	Total	18	100.0	19	100.0			
Vertical relationship	Malocclusion in vertical relationship	4	22.2	4	21.1	1.07	0.22–5.13	1.00
	Normal	14	77.8	15	78.9			
	Total	18	100.0	19	100.0			
Sagittal relationship	Malocclusion in sagittal relationship	5	27.8	4	25.0	1.15	0.25–5.33	1.00
	Normal	13	72.2	12	75.0			
	Total	18	100.0	16	100.0			
Presence of malocclusion	Any type of malocclusion	6	33.3	7	43.8	0.64	0.16–2.58	0.725
	Normal	12	66.7	9	56.3			
	Total	18	100.0	16	100.0			

It was observed in Table 5 that exclusive breastfeeding had a protective effect and diminished the risk of acquiring non-nutritive sucking habits OR 0.18 (CI 0.07–0.40, *p* = 0.001). Exclusive breastfeeding may have reduced the risk of pacifier use OR 0.24 (CI 0.11–0.52, *p* = 0.001). However, there was no significant association between exclusive breastfeeding and finger-sucking habit (*p* = 0.374). In addition, there was no significant association with intensity and duration of non-nutritive sucking habits.

**Table 5:** Relationship between exclusive breastfeeding and non-nutritive sucking habits

		Exclusive breastfeeding								
		Yes		No		Total				
		*N*	%	*N*	%	*N*	%	OR	CI 95 %	*p* value
Pacifier	Yes	14	50.0	200	81.0	214	77.8	0.24	0.11–0.52	0.001
	No	14	50.0	47	19.0	61	22.2			
	Total	28	100.0	247	100.0	275	100.0			
Finger sucking	Yes	0	0.0	14	5.7	14	5.1	0	NA	0.374
	No	28	100.0	233	94.3	261	94.9			
	Total	28	100.0	247	100.0	275	100.0			
Duration	>12 months	14	100.0	204	97.1	218	97.3	Infinity	NA	1
	<12 months	0	0.0	6	2.9	6	2.7			
	Total	14	100.0	210	100.0	224	100.0			
Intensity	Moderate or high use	5	41.7	132	64.4	137	63.1	0.39	0.12–1.29	0.131
	Low use	7	58.3	73	35.6	80	36.9			
Non-nutritive sucking habits	Yes	14	50.0	210	85.0	224	81.5	0.18	0.07–0.40	0.001
	No	14	50.0	37	15.0	51	18.5			
	Total	28	100.0	247	100.0	275	100.0			

## Discussion

The results in the present study indicated that there was no significant relationship between exclusive breastfeeding and the presence of any type of malocclusion. The results also indicated that there was no significant relationship between bottlefeeding and the presence of any type of malocclusion. However, it was observed that exclusive breastfeeding had a protective effect and diminished the risk of acquiring non-nutritive sucking habits.

At present, there is no consensus related to the association between feeding habits and malocclusion. Previous studies have reported that there was influence of feeding patterns on the development of malocclusion [1–8, 15–20]. According to some authors, breastfeeding and bottlefeeding involve different orofacial muscles, which possible have different effects on the harmonic growth of maxilla and dental arches. Meanwhile, breathing, swallowing, and mastication should be developed in harmony, and differences exist in the learning of the coordinated movement between breastfeeding and bottlefeeding children [18].

On the other hand, some studies did not found this association [11, 21, 22]. Some authors observed among children with minimal non-nutritive habits that those who breastfed had similar dental arch parameters and occlusal characteristics as those with shorter duration of breastfeeding or no breastfeeding [11]. A possible explanation is that the effects of breastfeeding on dental arch development are difficult to assess because it is not easy to separate these effects from those non-nutritive sucking behaviors. This result is because most breastfed children also engaged in at least some non-nutritive sucking.

The finding that exclusive breastfeeding had a protective effect and diminished the risk of acquiring non-nutritive sucking habits is in agreement with previous studies [6, 23–26]. In this investigation, thumb sucking has the same result with breastfeeding and bottlefeeding but the use of a pacifier was more utilized for bottlefeeding children. It is important to emphasize that breastfeeding seems to have non-nutritive protective impact. If the parents refuse to give the pacifier, the result may be different and thumb-sucking habit is something that children choose or need to do without the factor of parents giving the artifact to stimulate non-nutritive sucking.

A previous study investigated the influence of thumb sucking and pacifier use on breastfeeding patterns in exclusively breastfed infants, on the duration of exclusive breastfeeding, and on the total breastfeeding duration. The authors concluded that thumb sucking was clearly not related to the negative effects on the pattern and duration of breastfeeding. The possible negative effects of pacifier use on breastfeeding pattern and duration were related to frequency. In addition, occasional pacifier use was not found to have any negative effect on breastfeeding duration [27].

In offering an explanation for the relationships between breastfeeding and the risk of acquiring non-nutritive sucking habits, some potential limitations of this study should be taken into account. First, no cause-effect relationship can be deduced from a cross-sectional design study such as this one. Longitudinal designs (cohort studies) would increase the knowledge on this subject.

Moreover, there is much controversy surrounding the issue of diagnosis of malocclusions due to the inexistence of a universally accepted index. Comparisons between studies should be interpreted with caution due to the lack of uniformity in sample selection, diagnostic criteria, and classifications and age groups.

A positive attribute of this study was that children with non-nutritive sucking habits were excluded from the analysis. Recently, a systematic review also reported that substantial methodological and clinical heterogeneity was found among the studies in this issue. The major limitation is the failure to report controlling for confounder variables. Pacifier use and thumb sucking are confounder variables, since it is not possible to determine whether malocclusion was caused by bottlefeeding or pacifier/thumb sucking. The absence of controlling for confounders may have led to bias results in previous studies [10].

Some authors emphasized that breastfeeding alone seems not to be directly associated with malocclusions, but it may have a synergetic effect with parafunctional oral habits on the development of occlusofacial problems [28]. They also reinforced the arguments for the prevention of bad oral habits, especially among children who have not been fed at their mother’s breast or were breastfed for a short period.

Despite the multiple benefits of maternal breastfeeding, artificial feeding is widely used and may have contributed to the high rates of pacifier use and other harmful habits. Exclusive breastfeeding for at least 6 months of age is still the best recommendation to benefit children regarding their systemic health and harmonic craniofacial growth [10].

## Conclusions

In conclusion, there was no association between feeding habits and malocclusions in the deciduous dentition in this sample of children. Exclusive breastfeeding reduced the risk of acquiring non-nutritive sucking habits.
